# RPS9 promotes the progression of NSCLC via activation Stat3 and Erk signaling pathways

**DOI:** 10.7150/jca.67513

**Published:** 2022-02-14

**Authors:** Yiru Kong, Deng Shuangshuang, Xiaohua Liang, Xinli Zhou

**Affiliations:** 1 Department of Oncology, Huashan Hospital Fudan University, 12 Middle Urumqi Road, Shanghai 200040, China.; 2 Department of Oncology, Shanghai Medical College Fudan University, Shanghai, 200032, China.; 3 Department of Coronary Care Unit, The East Division of the First Affiliated Hospital, Sun Yat-sen University, Guangzhou, 510700, China.

**Keywords:** Non-small cell lung cancer, RPS9, progression, Stat3 and Erk signaling pathways

## Abstract

**Background**: Non-small cell lung cancer (NSCLC) accounts for the largest pathological type of lung cancers, and it is characterized by high incidence and poor prognosis. However, the molecular mechanisms involved in development and progression of NSCLC are not well elucidated. In this study, we aimed to explore the role and regulatory mechanism of RPS9 in NSCLC.

**Materials and methods**: The RPS9 expression in NSCLC tissues and cell lines was assessed by qRT-PCR and western blot. Knockdown of RPS9 induced by RNA interference (RNAi) method in PC9, A549 and H1299 cells. Overexpression of RPS9 induced by transient transfection in H292 cells. Cell proliferation, colony formation, metastasis and apoptosis abilities were determined by CCK-8 assay, colony formation assay, transwell assay and flow cytometry, respectively. The host signaling pathways affected by RPS9 were screened by antibody library and proved by western blot.

**Results**: RPS9 was significantly upregulated in NSCLC tissues and cell lines. Overexpression of RPS9 predicted poor prognosis of NSCLC patients. Knockdown of RPS9 obviously repressed cell proliferation, metastasis, and induced apoptosis. Mechanistically, suppression of RPS9 inhibited the expression level of phosphorylation of Stat3 and Erk.

**Conclusion**: Our study clarified that knockdown of RPS9 inhibits the progression of NSCLC via inactivation Stat3 and Erk signaling pathways.

## Introduction

Lung cancer is one of the most frequently diagnosed malignant cancer and the first cause of cancer-related death in the world 1, 2, with 2.09 million new cases and 1.76 million deaths worldwide in 2018 3. NSCLC accounts for 75-80% of all pathological type of lung cancers. Although great improvements have been made in screening of people at high risk, advancements in research and drug development and systemic therapies (such as surgical resection, personalized biomarker-driven treatment, immunotherapies, and chemo-radiation treatments), most NSCLC patients are diagnosed at an advanced stage and their prognosis is unsatisfactory 4. NSCLC remains a highly lethal disease and the overall 5-year survival rate of NSCLC patients is less than 25% 5. Thus, it is crucial to expand our knowledge of new biomarkers and molecular basis of carcinogenesis for early diagnosis, intervention, and treatment to reduce the mortality of NSCLC.

RPS9 is ribosomal protein gene and located at chromosome 19ql3.4 6. RPS9 protein directly binds to the 18S rRNA and plays an essential role in ribosome biogenesis and stabilization, DNA repair and developmental regulations, mRNA unwinding, decoding accuracy and malignant transformation 7-10. For example, Pnueli et al. demonstrated that RPS9 participated in the regulation of mRNA translation and possibly translation termination 11. Studies have shown that RPS9, as a novel B23/ NPM binding protein, is necessary for normal cell growth and proliferation 12.

Previous studies have suggested that RPS9 dysregulation was involved in tumorigenesis, such as pancreatic cancer, head, and neck cancer (such as head and neck squamous cell carcinoma) and so on 13-15. Moreover, evidence indicated suggested that RPS9 knockdown suppressed proliferation through P53 independent mechanisms in U2OS cells, impaired 18S rRNA production in glioma cells by P53 and accelerated cell death in cervical carcinoma HeLa cells 16. In this context, RPS9 is involved in tumorigenesis in both positive and negative ways depending on different tumor type and regulatory mechanism. The role of RPS9 in NSCLC was previously revealed, but the function and related regulatory mechanism of RPS9 in the development of NSCLC remain obscure.

In this research, we provide evidence for the first time that RPS9 is upregulated in NSCLC patients and cell lines and overexpression of RPS9 indicates poor prognosis. Strikingly, RPS9 knockdown inhibits NSCLC cell proliferation, colony formation and metastasis. In addition, RPS9 suppressed apoptosis in NSCLC cells. More interestingly, we found that knockdown of RPS9 could inhibit the progression of NSCLC via regulating Stat3 and Erk signaling pathways. This study highlights the function of RPS9 in NSCLC growth and metastasis and may facilitate development of new diagnostic or therapeutic biomarker to improve the outcome of NSCLC patients.

## Materials and methods

### NSCLC tissues and cell culture

A total of 143 pairs of primary NSCLC and matched adjacent normal lung tissues were collected from patients who underwent surgery between 2017 and 2019 in Huashan Hospital, Fudan University. Written informed consents were signed before the research started. And this research was authorized by the Ethics Committee of Huashan Hospital, Fudan University. All procedures were carried out in accordance with the ethical guidelines of the Declaration of Helsinki. Three NSCLC cell lines (PC9, A549 and H1299) and normal human bronchial epithelial cells (16HBE) were obtained from the Cell Bank of the Chinese Academy of Sciences (Shanghai, China). All the cells were cultured in Dulbecco's Modified Eagle Medium (DMEM Gibco, USA) supplemented with 10% fetal bovine serum (FBS, Gibco, USA) and 1% penicillin-streptomycin (Invitrogen, USA) at 37 ºC. Cells were used when they were in the logarithmic growth phase.

### RNA isolation and quantitative Real-Time polymerase chain reaction (qRT-PCR) assays

The total RNA from NSCLC tissues and cell lines was purified using RNAiso Plus reagent (Invitrogen, USA). Then, RNA was converted to complementary DNA (cDNA) by the PrimeScript RT Reagent Kits (TaKaRa, China) according to the manufacture's protocol. The qRT-PCR assays were carried out using SYBR Premix Ex Taq II Kit (TaKaRa, China). The following primer sequences were used: RPS9: Forward: 5'-GAAGCGGAGCCAACATGC-3', Reverse: 5'-ATACTCGCCGATCAGCTTCAG-3'. GAPDH served as an internal control.

### Cell transfection

The siRNAs targeting RPS9, and corresponding negative control siRNAs (si-NC) were designed and synthesized by Genechem (Shanghai, China). Plasmid used for transient transfection were designed and synthesized by Ribobio (Shanghai, China). Cells were plated at 60-70% confluence in a 6-well plate. Cell transfection was performed using Lipofectamine 2000 reagents (Invitrogen, USA). After transfection for 48 hours, the knockdown and overexpression efficiency were validated by both qRT-PCR and western blot.

### Transwell migration and invasion assays

For transwell migration assays, cells were resuspended in DMEM without FBS and 4 × 10^5 cells were seeded into the upper chamber of a 24-well insert (Corning Inc., USA) precoated with Matrigel. 800-µL DMEM supplemented with 10% FBS was placed in the lower chamber. After incubation for 24 hours, the invaded cells on the lower surface were fixed with methanol, stained with crystal violet and the number of invaded cells from five random fields was counted under a light microscope. For the invasion assay, 1 × 10^6 cells were seeded into the upper Matrigel invasion chambers (Corning BioCoat, USA), and the following procedures were carried out similarly as what was described in the transwell migration assays.

### Cell viability and colony assay

Cells were seeded into 96-well plates in triplicate and cell viability was detected by the Cell Counting Kit-8 (CCK-8) assay (Dojindo, Japan) according to the manufacturer's instructions. For colony formation assay, cells were plated in 6-well plates (1000 cells/well) and cultured for 14 days. After washed with phosphate-buffered saline (PBS) for three times, the colonies were fixed in methanol and subsequently stained with crystal violet for 30 min.

### Cell cycle analysis and apoptotic assay

For cell cycle analysis, after washed with PBS for three times, cells were collected and fixed with 70% ethanol. Following PBS washing, the cells were stained with 500μl propidium iodide (PI) (Beyotime, China) contained RNaseI (Beyotime, China) according to the manufacturer's instructions. Subsequently, the cell cycle was analyzed by a FACSCalibur flow cytometer (BD Biosciences, USA). For apoptotic assay, after washed with PBS for three times, cells were stained with Annexin V-FITC and PI (Beyotime, China) at room temperature for 30 min. Then, the apoptotic cells were assessed using flow cytometer.

### Protein extraction and western blot

Protein lysate were extracted using RIPA lysis buffer (Thermo Fisher Scientific, China) contained protease and phosphatase inhibitors (1:100, Thermo Fisher Scientific, China). Subsequently, proteins were electrophoresed by 10% sodium dodecyl sulfate polyacrylamide gel electrophoresis (SDS-PAGE) and transferred onto polyvinylidene fluoride membranes (PVDF, Millipore, Billerica, USA). After blocked with 5% bovine serum albumin for 1 hour, the membranes were incubated with following primary antibodies at 4℃ overnight: RPS9 (Proteintech, 1:1000); P- mTOR, mTOR , P-FAK, FAK, P-FoxO3a, FoxO3a, P-FoxO1, FoxO1, P-Stat3, Stat3, NF-κB, P-AMPK, AMPK, P-Smad2, Smad2, P-AKT, AKT, TGF-β, P-Erk and Erk (1:1000, Cell Signaling Technology, USA); β-actin (1:10000, Sigma-Aldrich, USA). Next day, after washed in PBST for three times, the membranes were incubated with HRP-conjugated anti-mouse or anti-rabbit antibodies (1:5000, Sigma-Aldrich, USA) at room temperature for 1 hour. Finally, the bands were visualized and analyzed by SuperSignal West Femto Maximun Sensitivity Substrate (Thermo Fisher Scientific, USA).

### Statistical analysis

The results were presented as mean ± SEM (Standard error of mean). The unpaired two-tailed Student's t-test was used to compare the differences between two groups and the data were visualized by Graphpad prism version 8.0 software. All statistical analysis was conducted with SPSS version 22.0 software. A p-value less than 0.05 was considered statistically significant.

## Results

### RPS9 is highly expressed in NSCLC tissues

**Figure 1:**
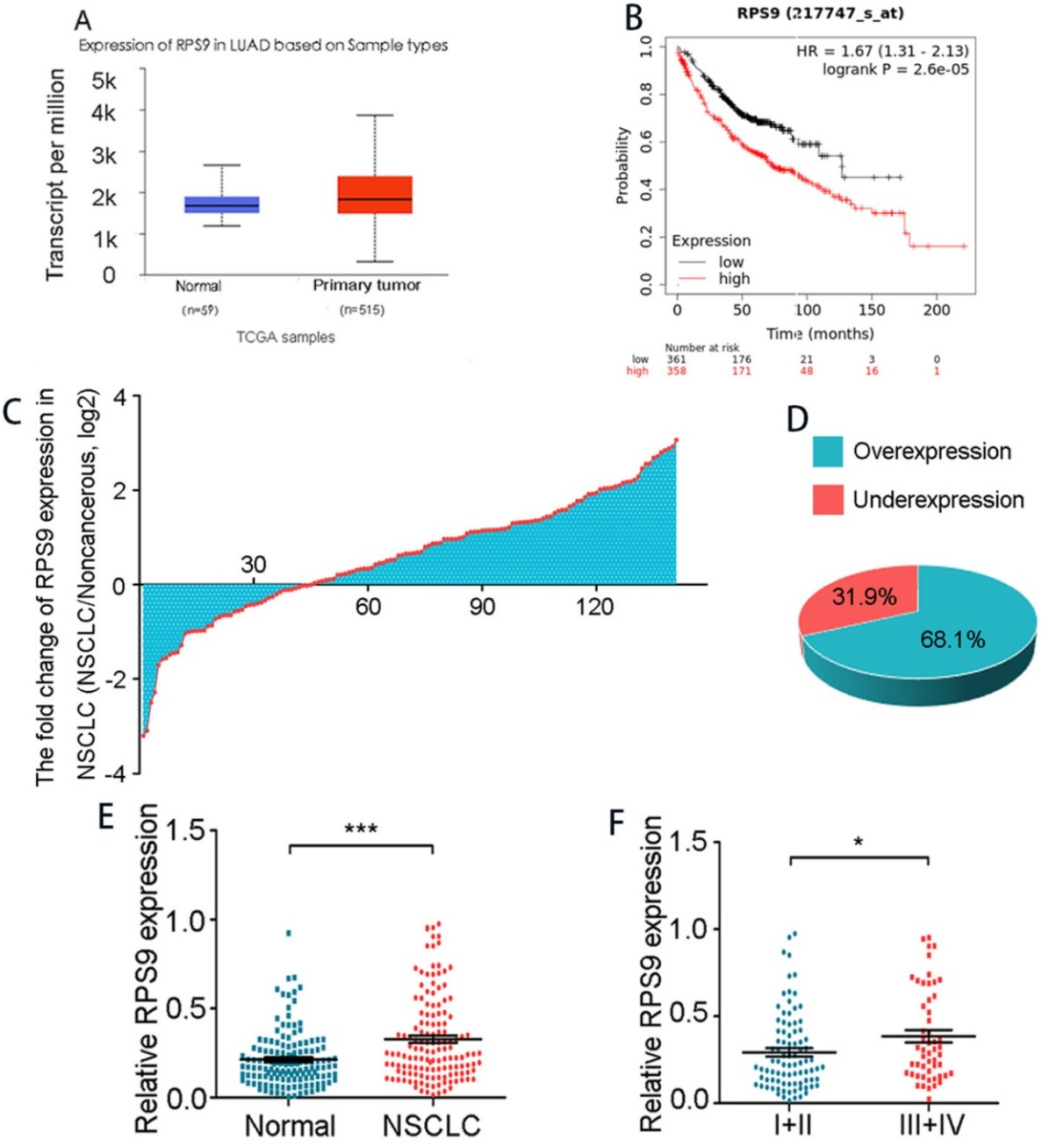
** RPS9 is highly expressed in NSCLC tissues. (A)** RPS9 was over-expressed in adenocarcinoma samples from TCGA database.** (B)** Kaplan-Meier plot analyses of the correlation between RPS9 expression and overall survival of NSCLC patients in the GEO cohort.** (C)** Relative RPS9 mRNA expression in 141 NSCLC patients by qRT-PCR.** (D)** RPS9 was overexpressed in 68.1% NSCLC patients.** (E)** Relative RPS9 mRNA expression in 141 pairs of primary NSCLC and matched adjacent normal tissues.** (F)** Relative RPS9 mRNA expression in different clinical stage. *p < 0.05; **p < 0.01; ***p < 0.001.

To examine the potential role of RPS9 in NSCLC tissue, we firstly analyzed RPS9 mRNA levels in NSCLC data from TCGA database via UALCAN, the results confirmed that RPS9 was highly expressed in primary adenocarcinoma samples compared with normal samples (Figure 1A). Kaplan-Meier plot survival analysis based on GEO database showed that overexpression of RPS9 was associated with poor outcomes in NSCLC patients (Figure 1B). Subsequently, a total of 141 pairs of primary NSCLC and matched adjacent normal tissues were collected to further clarify these results in NSCLC, finding that RPS9 was extremely overexpressed in most NSCLC tissues compared to normal controls (Figure 1C) and this phenomenon was determined in 68.1% of NSCLC cases (Figure 1D). Moreover, we carried out the qRT-PCR to test the RNA level of each sample and the results reveal that the expression level is positively correlated with advanced TNM stages (Figure 1F). However, no significant difference was observed between the expression level of RPS9 and age, gender, tumor size, differentiation, and metastasis (Table 1). Taken together, the results identify that RPS9 is up expressed in NSCLC tissues and overexpression of RPS9 indicates poor prognosis.

### RPS9 is up regulated in NSCLC cell lines

**Figure 2:**
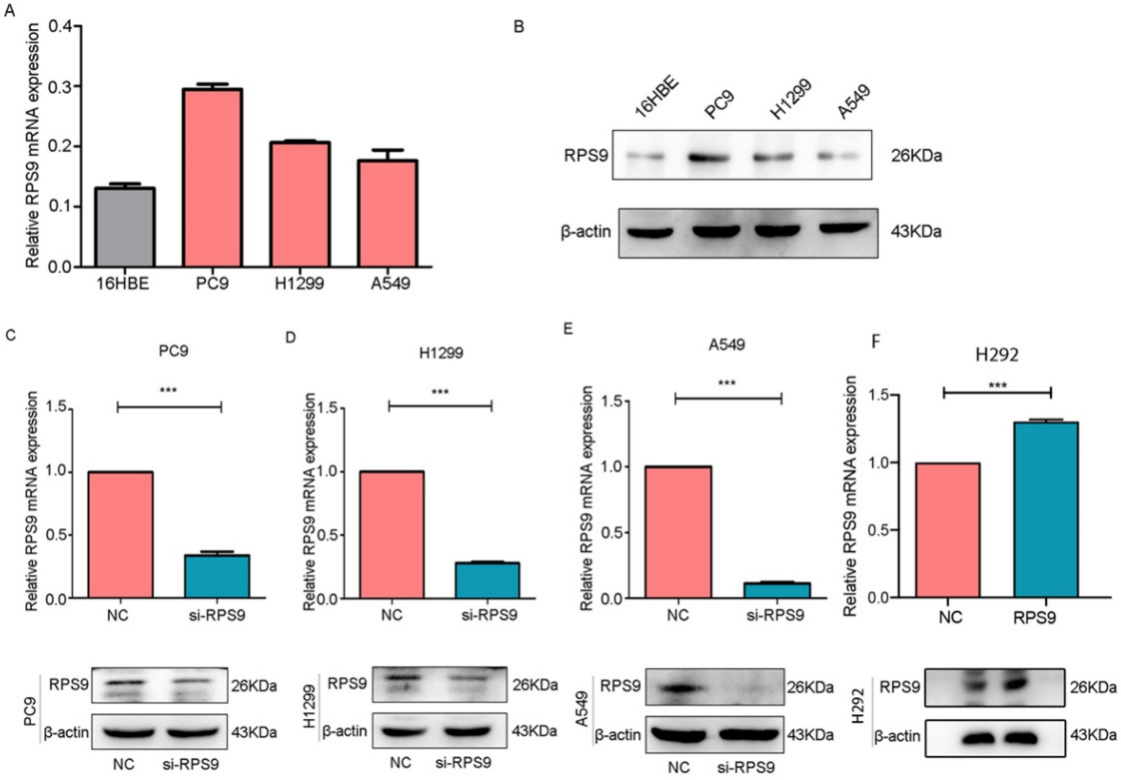
** RPS9 is up regulated in NSCLC cell lines. (A)** and **(B)** Relative mRNA and protein expression level of RPS9 in three NSCLC cell lines (PC9, A549 and H1229) and normal human bronchial epithelial cells 16HBE. **(C-E)** The knockdown efficiency of cells after transfection with si-RPS9 in PC9, A549 and H1229 cell lines, respectively. **(F)** The overexpression efficiency of cells after transfection with plasmids in H292 cell line. *p < 0.05; **p < 0.01; ***p < 0.001.

To identify the biological function of RPS9 in NSCLC, we assessed the RPS9 expression levels in three NSCLC cell lines (PC9, A549 and H1299) and the normal human bronchial epithelial cells (16HBE) by qRT-PCR and immunoblotting analysis. As shown in Figure 2A and 2B, the expression of RPS9 was relatively higher in NSCLC PC9 and H1299 cell lines slightly higher in A549 cell lines than that in normal bronchial epithelial 16HBE cell line. Together, the data above identify that RPS9 is up expressed in NSCLC cell lines.

### RPS9 exerts tumor-stimulative functions of NSCLC cells

**Figure 3:**
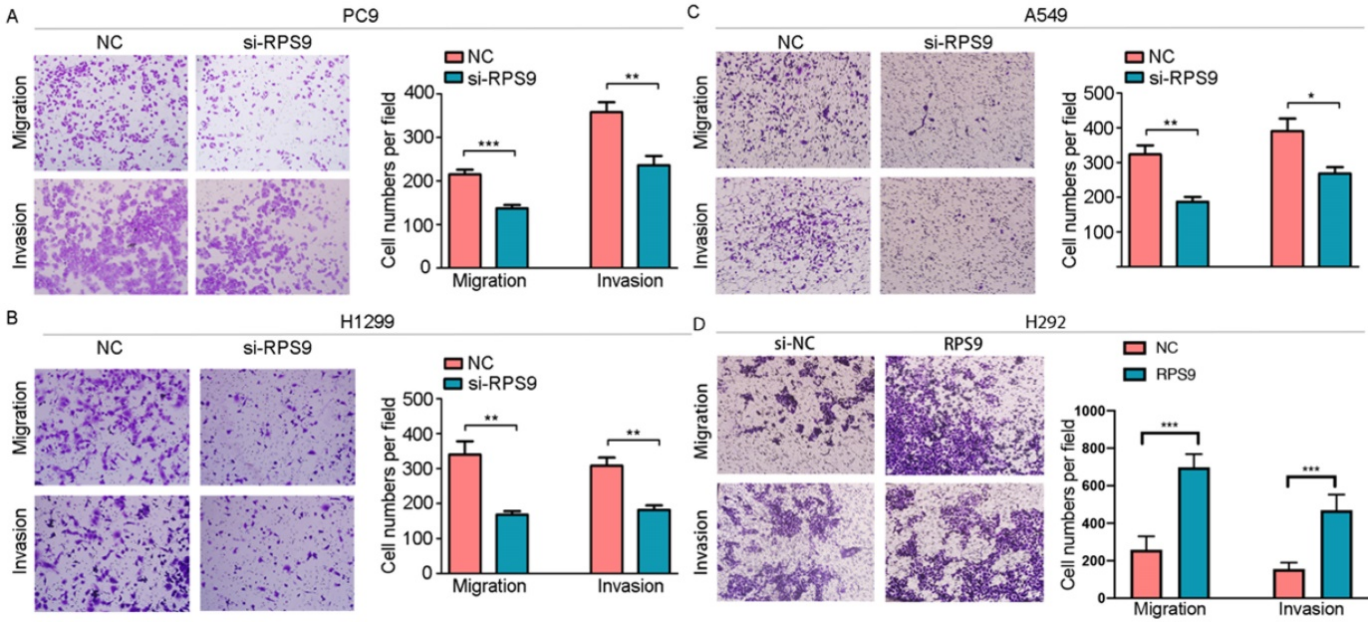
** Effect of RPS9 on invasion and migration of lung cancer cells* in vitro*. (A-D)** Effects of RPS9 on migratory and invasive capacity of PC9, A549, H1299, and H292 cell lines were detected by transwell migration and invasion assays. *p < 0.05; **p < 0.01; ***p < 0.001.

**Figure 4:**
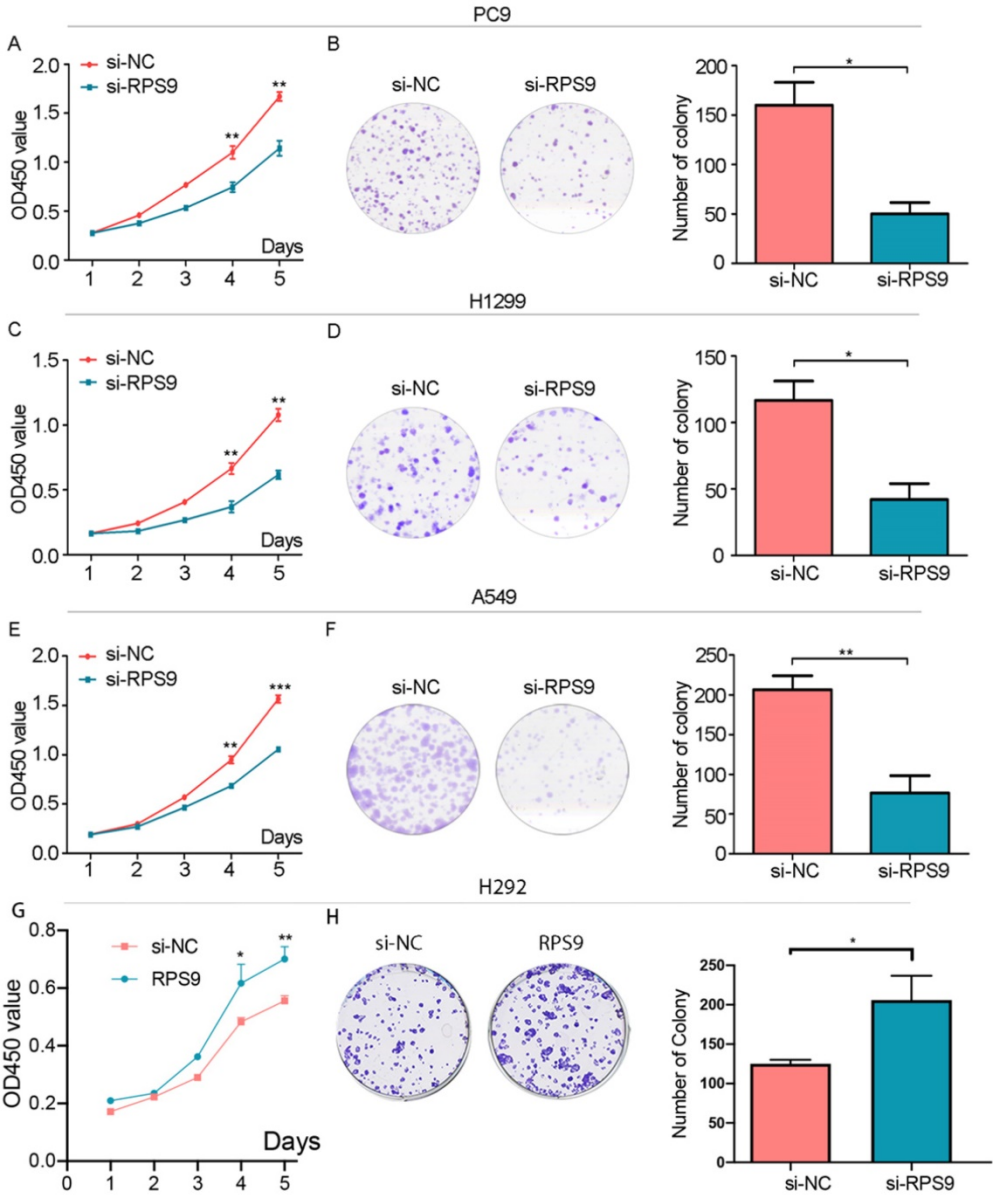
** Effect of RPS9 on clonal formation of lung cancer cells *in vitro*. (A)**, **(C)**, **(E)** and **(G)** Effects of RPS9 on proliferative abilities of PC9, A549, H1299 and H292 cell lines were detected by CCK-8 assay. **(B)**, **(D)**, **(F)** and **(H)** Effects of RPS9 on cell viability of PC9, A549, H1299 and H292 were measured by colony formation assay. *p < 0.05; **p < 0.01; ***p < 0.001.

**Figure 5:**
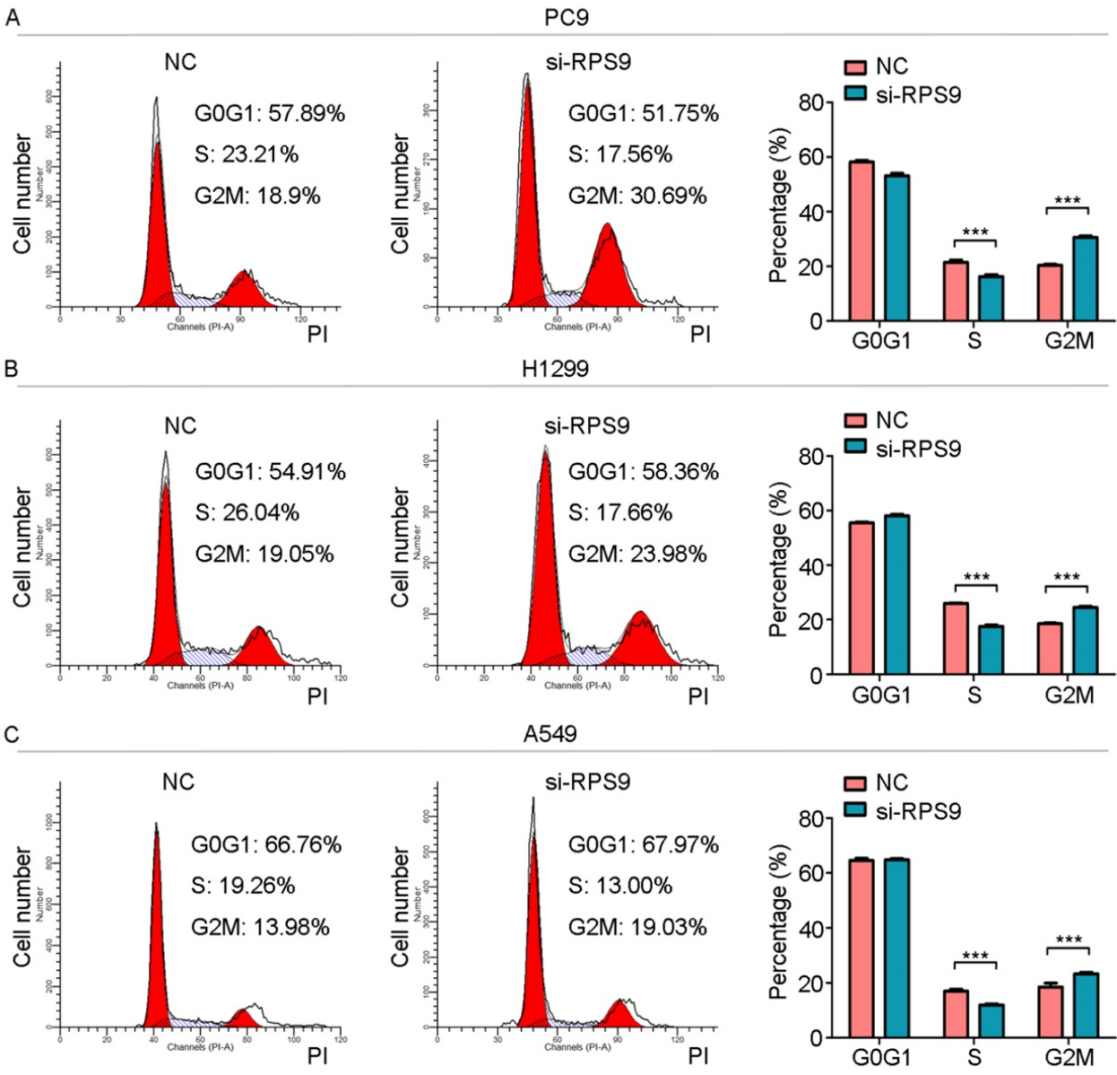
** RPS9 promotes proliferation of NSCLC cells. (A-C)** Flow cytometric analysis showing the effects of RPS9 knockdown on cell cycle of PC9, A549 and H1229 cells. ***p < 0.001.

To investigate the impact of RPS9 in NSCLC, we next silenced RPS9 expression in PC9, A549 and H1299 cell lines using si-RNA. The effective knockdown efficiency was validated by both qRT-PCR and immunoblotting analysis, demonstrating that RPS9 was significantly inhibited in PC9, A549 and H1299 cell lines after transfection with si-RPS9 (Figure 2C-E). We also overexpressed RPS9 in H292 cell lines and the efficiency of RPS9 overexpression was validated by qPCR and western blotting assay (Figure 2F). Knockdown of RPS9 significantly inhibited the proliferation and colony formation abilities of NSCLC cells compared with control cells, while overexpression of RPS9 substantially promoted the proliferation and colony formation abilities of NSCLC cells compared with control cells. Then, transwell migration and invasion assays were carried out to assess the impact of RPS9 on invasive and metastatic phenotype of NSCLC (Figure 3A-C). The results revealed that knockdown of RPS9 inhibited cell migratory and invasive capacity. On the contrary, overexpression of RPS9 significantly increased the invasion and migratory abilities of H292 cells (Figure 3D). In addition, CCK-8 and colony formation assays showed that knockdown of RPS9 suppressed the cell proliferation (Figure 4A, 4C and 4E) and cell colony formation abilities (Figure 4B, 4D and 4F). The cell cycle was detected by flow cytometry. Compared with the control group, RPS9 silence resulted in an arrest in G2/M phase in PC9, A549 and H1299 cell lines (Figure 5A, 5B and 5C, respectively). However, no significant difference was observed in groups in G0 and G1 phases of cell cycle after RPS9 suppression. Collectively, these results show that RPS9 accelerates NSCLC migration, invasion, and proliferation capacities *in vitro*.

### RPS9 inhibits apoptosis of NSCLC cells

**Figure 6:**
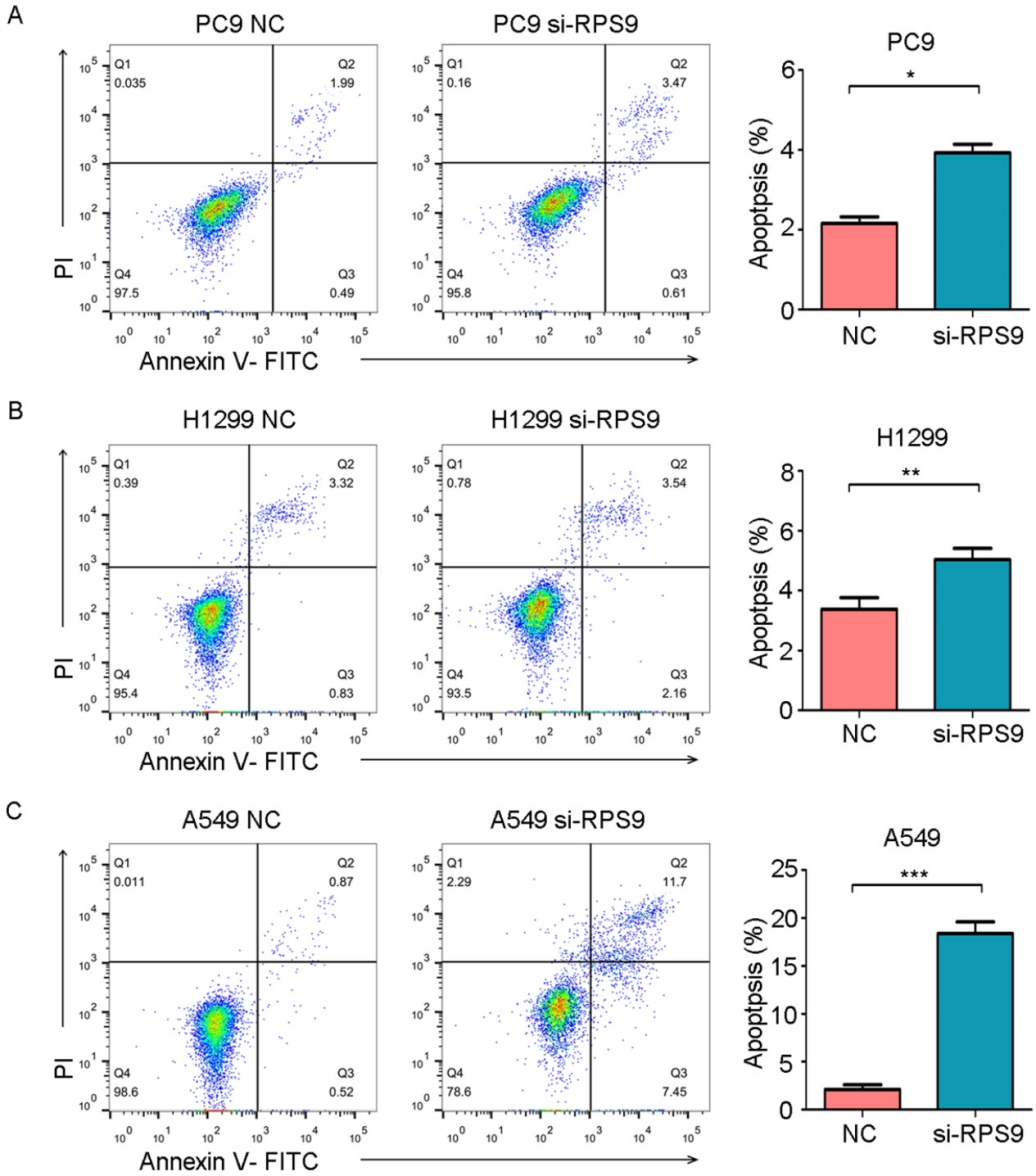
** RPS9 inhibits apoptosis of NSCLC cells. (A-C)** Flow cytometric analysis showing the effects of RPS9 knockdown on cell apoptosis of PC9, A549 and H1229 cells. *p < 0.05; **p < 0.01; ***p < 0.001.

To further examine whether RPS9 affects cell apoptosis capacities of NSCLC, flow cytometry was also utilized to determine the percentage of cell apoptosis. As shown in Figure 6, the cell apoptosis rates of PC9, A549 and H1299 were obviously increased after RPS9 knockdown. Thus, RPS9 not only promotes growth and metastasis of NSCLC cells but also suppresses cell apoptosis.

### RPS9 facilitates NSCLC progression by regulating Stat3 and Erk signaling pathways

**Figure 7:**
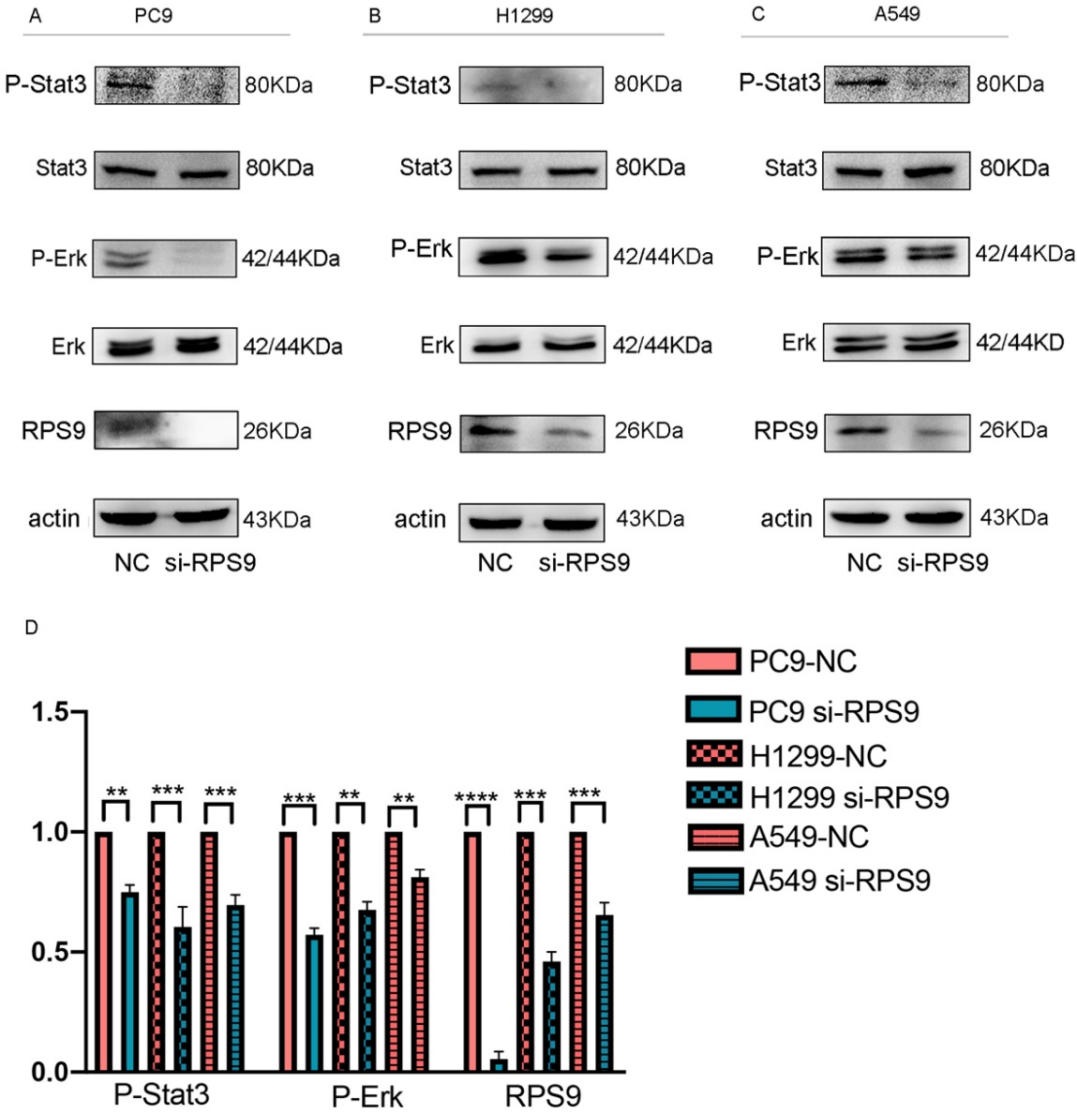
** RPS9 facilitates NSCLC progression by regulating Stat3 and Erk signaling pathways. (A-C)** Western blot analyzed the relative phosphorylated protein and total protein levels in PC9, A549 and H1229 cells transfected with si-NC and si-RPS9.** (D)** Western blot analysis of the phosphorylated protein levels of P-Stat3, P-Erk, and RPS9 in PC9, A549 and H1229 cells transfected with si-NC and si-RPS9.

Seeking to define the potential mechanism of RPS9 promoting NSCLC progression, the common phosphorylated protein related antibodies and their total protein antibodies including P- mTOR, mTOR , P-FAK, FAK, P-FoxO3a, FoxO3a, P-FoxO1, FoxO1, P-Stat3, Stat3, NF-κB, P-AMPK, AMPK, P-Smad2, Smad2, P-AKT, AKT, TGF-β, P-Erk and Erk from antibody library were utilized. Subsequently, the above phosphorylated protein and total protein levels were examined by western blot. As shown in Figure 7, the results argued that RPS9 knockdown inhibited the phosphorylation of P-Stat3 and P-Erk. However, no significant difference was detected in the total proteins Stat3 and Erk and other phosphorylated protein (supplementary file). These results indicate that RPS9 accelerates NSCLC progression by modulating Stat3 and Erk signaling pathways.

## Discussion

Ribosomal proteins, as indispensable components of living cells, are long considered as highly conserved and exert diverse roles in different cells 17. Recently, ribosomal proteins have gained much attention in malignant transformation 18. And a growing number of researches suggest that both increased numbers and altered modifications of ribosomes drive tumorigenesis by decreasing translation fidelity or changing mRNA translation and other mechanisms 19. For example, RPS3, a component of the 40S ribosomal submit, accelerates hepatocarcinogenesis by post transcriptionally up-regulating SIRT1 20. Additionally, Lim et al. reported that RPS3a is upregulated in HBV-associated hepatocellular carcinoma and promotes hepatocarcinogenesis by enhancing Hepatitis B virus X protein (HBx)-induced NF-kB signaling pathway 21. Previous work proved that RPS6 acted as anti-HER2 drug-resistant factors in HER2-amplified gastric cancer 22. Moreover, increased expression of ribosomal proteins RPS11 and RPS20 predicts shorter survival in glioblastoma patients 23. What's more, RPS9 can also exerts tumor stimulative functions in Osteosarcoma through Inactivation of MAPK Signaling Pathway^24^, which gives us more evidence that RPS9 may play a promoting role in tumorigenesis.

Previously, RPS9 could act as a tumor suppressor in breast cancer, pancreatic cancer, and glioma 13, 25. Specifically, overexpression of RPS9 predicts lower death rate in glioma, probably via P53 activation and leading to suppression of cell proliferation. Similarly, Fang et al. reported that downregulation of RPS9 was associated with worse overall survival of patients with breast cancer 26, while upregulation of RPS9 was associated with improved long term survival of breast cancer, probably by regulation of DNA methylation 27.

Reversely, we revealed that RPS9, as a member of ribosomal proteins, was significantly up regulated in NSCLC tissue samples compared to the corresponding non-cancerous tissues. In addition, high levels of RPS9 in NSCLC tissues are related to poor prognosis. In the present study, RPS9 knockdown suppressed tumor growth, metastasis and promoted cell apoptosis, which is consistent with the previous researches in osteosarcoma, glioma and cervical carcinoma 16. Previously, RPS9 has been reported to have an oncogenic role in colorectal cancer, neuroblastoma cells and NSCLC 28-30. Wenhan Yang et.al found that BRCAT54 directly bound to RPS9. Knockdown of RPS9 substantially reversed the promoting effects of si-BRCAT54 on cell proliferation and enhanced the inhibitive effect of si-BRCAT54 on BRCAT54 expression. In addition, silencing of RPS9 activated JAK-STAT pathway and suppressed calcium signaling pathway gene expressions^28^. What's more, RPS9 has aberrant promoter hypermethylation and was involved in colorectal tumorigenesis 29. Other than that, knockdown of RPS9 induced G2/M arrest of colon cancer cells by downregulating CDK1 expression at the promoter level and dysregulation of RPS9 may be involved in microsatellite instability of colorectal cancer 31] [32.

In NSCLC, it has been reported that RPS9 was involved in the activation of JAK-STAT and calcium signaling pathways. In the previous study, knockdown of RPS9 offset the promoting effects of si-BRCAT54 on cell proliferation and silencing of RPS9 activated JAK-STAT pathway and suppressed calcium signaling pathway gene expressions 28. However, whether RPS9 accelerates the pathogenesis of NSCLC via any other mechanisms still needs to be explored. In this study, to further investigate the underlying mechanisms of RPS9 involved in NSCLC progression, antibody library including common phosphorylated protein related antibodies and their total protein antibodies was performed. We examined the total protein levels by western blot, and the results demonstrated that suppression of RPS9 inhibited the phosphorylation of Stat3 and Erk.

In conclusion, our findings exposed that RPS9 acted an oncogene in NSCLC via regulating the phosphorylation expression of Stat3 and Erk, promoting cell proliferation, migration, invasion, and inhibiting apoptosis in NSCLC. The role of RPS9 on the occurrence and metastasis of lung cancer needs more research. With the emergence of more studies, RPS9 may serve as an important marker for clinical prediction and diagnosis.

## Author Contributions

Yiru Kong involved in data curation, methodology, writing—review and editing. Shuangshuang Deng involved in formal analysis, investigation, writing—review and editing. Xiaohua Liang involved in resources, writing—review and editing. Xinli Zhou involved in conceptualization, resources, writing—review and editing.

## Figures and Tables

**Table 1:** Relationship between RPS9 expression and clinicopathologic characteristics in 141 NSCLC patients.

Characteristics	Number of cases (%)	RPS9 expression	
		Mean± SD	P value
Age			
≤60	53 (37.6)	0.362±0.243	0.236
>60	88 (62.4)	0.308±0.236	
Gender			
Female	54 (38.3)	0.362±0.241	0.183
Male	87 (61.7)	0.307±0.236	
Tumor size(cm)			
≤ 3	79 (56.0)	0.329±0.255	0.956
>3	62 (44.0)	0.327±0.219	
Tissue			***
NSCLC	141	0.328±0.240	0.000
Noncancerous	141	0.214±0.155	
Pathological type			***
Adenocarcinoma	103 (73.0)	0.371±0.255	0.000
Squamous cell carcinoma	38 (27.0)	0.211±0.137	
Degree of differentiation			
I-II	68 (48.2)	0.295±0.203	0.114
III	73 (51.8)	0.376±0.266	
Clinical stage			*
I+II	90 (63.8)	0.293±0.220	0.021
III+IV	51 (36.2)	0.390±0.262	
Metastasis			
No	82 (58.2)	0.323±0.224	0.090
Yes	59 (41.8)	0.369±0.254	

P-value represents the significant differences using Student's t-test between variable subgroups (*P<0.05, **P<0.01 and ***P<0.001). The bold formatting used in the table was considered to have a significant difference
